# The Hotspots and Publication Trends in Central Venous Catheter‐Related Bloodstream Infection: A Bibliometric Analysis

**DOI:** 10.1111/nicc.70596

**Published:** 2026-08-06

**Authors:** Jianrong Li, Zhonghua Li, Leping Wang, Xuefang Liu, Junyu Zhu

**Affiliations:** ^1^ Department of Neurosurgery The Second Hospital of Hebei Medical University Shijiazhuang China; ^2^ Department of Critical Care Medicine Luquan Branch, The Second Hospital of Hebei Medical University Shijiazhuang China

**Keywords:** bibliometric analysis, bloodstream infections, central venous catheter, infection control, surveillance

## Abstract

**Background:**

Central venous catheter‐related bloodstream infections (CRBSIs) are a major healthcare‐associated infection, particularly in intensive care unit (ICU) settings.

**Aim:**

To conduct a bibliometric analysis of global research trends, collaborative networks and evolving hotspots in the field of CRBSIs from 1977 to 2024.

**Study Design:**

A comprehensive literature search was conducted using the Web of Science Core Collection (WoSCC) for publications from 1977 to 2024. Bibliometric tools, including VOSviewer, CiteSpace and the R package ‘bibliometrix’ were utilized to analyse research trends, collaboration networks and keyword dynamics.

**Results:**

A total of 4724 publications were identified, with an annual growth rate of 8.5%. The United States led in publication output (1788), followed by China and Italy. Harvard University and Johns Hopkins University were among the most influential institutions. Key journals included *American Journal of Infection Control* and *Infection Control and Hospital Epidemiology*. Notable authors, such as Victor D. Rosenthal and Jonathan R. Edwards, contributed significantly to the field, with high citation and h‐index metrics. Keyword co‐occurrence network analysis revealed that recent studies had concentrated on topics such as epidemiology and outcomes, treatment, prevention and guidelines for CRBSIs. Besides, since 2019, the keywords ‘infection control’ and ‘society’ and ‘guidelines’ had been still prominently concentrated.

**Conclusions:**

This bibliometric analysis highlights the sustained growth and global collaboration in CRBSIs research. Current research hotspots focus on the management of CRBSIs and the implementation of clinical guidelines.

**Relevance to Clinical Practice:**

This bibliometric analysis underscores the critical importance of implementing evidence‐based infection control bundles and adhering to updated clinical guidelines to prevent and manage CRBSIs. It highlights the pivotal role of nursing professionals in consistently executing preventive measures, such as hand hygiene and maximal sterile barrier precautions. Furthermore, the findings advocate for optimized diagnostic and treatment pathways, including prompt blood cultures and targeted therapy and emphasize the need for multidisciplinary collaboration to drive continuous quality improvement in critical care settings.

## Impact Statements

- What is known about the topic
  ○ CRBSIs represent a major healthcare‐associated infection with significant morbidity and mortality, particularly within intensive care unit settings.
  ○ Global research output in this field has expanded substantially over recent decades.
  ○ Established research foci include the epidemiology of CRBSIs, preventive strategies, treatment modalities and the implementation of clinical guidelines.
- What this paper adds
  ○ This paper provides a comprehensive bibliometric mapping of global CRBSIs research spanning nearly five decades, quantifying publication growth and international collaborative networks.
  ○ This paper identifies a distinct evolution in research hotspots, shifting from basic pathophysiology toward practical implementation science.
  ○ This paper highlights the increasing predominance of guideline adherence, infection control strategies and stewardship programmes as key areas of contemporary investigation.

## 1 Introduction

Central venous catheter‐related bloodstream infections (CRBSIs) are defined as bloodstream infections arising from the use of central venous catheters (CVCs), typically caused by microbial contamination at the catheter insertion site or within the catheter lumen [1, 2], contributing to considerable morbidity, mortality and economic burden worldwide. Approximately 80 000 CRBSIs cases occur annually in the United States, resulting in nearly 30 000 deaths, and a similarly high incidence is reported in China, with significant associated medical expenses [3, 4]. Effective management of CRBSIs requires an integrated strategy, including catheter removal, targeted antimicrobial therapy and infection prevention measures. Definitive diagnosis relies on blood cultures, which identify the causative pathogen and guide appropriate treatment [5]. The complexity of CRBSIs management is reflected in the growing body of research, yet the expanding volume of literature makes it difficult for researchers to identify overarching trends and evolving priorities.

The management and prognosis of CRBSIs are complex, requiring an integrated strategy involving catheter management, targeted antimicrobial therapy and prevention [6]. Removal of the catheter remains the primary intervention aimed at preventing further dissemination; however, in patients dependent on long‐term venous access—such as those receiving chemotherapy or parenteral nutrition via central venous catheters—antimicrobial lock therapy (ALT) can be used to preserve catheter function while controlling infection [7, 8]. Additionally, systemic antibiotic therapy tailored to the identified pathogen and local resistance patterns is also fundamental to treatment success [9, 10]. Poor outcomes are more common in older or immunocompromised patients with multiple comorbidities, whereas infections caused by multidrug‐resistant organisms or those resulting in severe complications carry higher mortality [11, 12]. Prompt, effective treatment significantly improves survival, while delayed or inadequate therapy worsens prognosis [13]. Given the rapidly expanding body of literature in this field, it has become increasingly challenging for researchers to identify macro‐level trends, underscoring the need for comprehensive bibliometric analysis.

Bibliometric analysis provides a novel and efficient method for quantitatively and qualitatively analysing publications [14]. Although research on the management and prevention of CRBSIs has accumulated steadily in recent years [15, 16, 17], a comprehensive bibliometric analysis utilizing visualization tools to identify and map research trends remains lacking. To address this knowledge gap, this bibliometric analysis aims to yield valuable insights that can inform future advancements in CRBSIs, attempting to identify research trends and potential hot spots in this field.

## 2 Methods

### 2.1 Search Strategies and Data Collection

A bibliometric analysis was conducted to investigate the research landscape of CRBSIs using the Web of Science Core Collection (WoSCC), an authoritative and multidisciplinary database. The search covered publications from 1 January 1977 to 17 December 2024.

The search formula was set as follows: (TS = (“central venous catheterization” OR “central line*” OR “central venous catheter*” OR “central vein catheter*” OR “central venous cannulation” OR “central venous access device*” OR “peripherally inserted central catheter*”)) AND TS = (bloodstream infection* OR septicemia* OR blood poisoning* OR Sepsis OR bacteremia*) [15, 16, 17]. The inclusion criteria for this study were: (1) publications focusing on CRBSIs, (2) original research articles, (3) articles published in English, and (4) articles published between 1 January 1977 and 17 December 2024. The exclusion criteria were: (1) publication types other than original articles, including letters, meeting abstracts, conference proceedings, editorial materials, early access publications and reviews, (2) Exclusion of literature not relevant to the research topic. To prevent deviations due to database updates, we conducted the literature retrieval on a single day (17 December 2024). All information was collected in text format, including the number of publications and citations, titles, author information, institutions, countries/regions, keywords and journals.

### 2.2 Statistical Analysis

The bibliometric analysis employed three tools: VOSviewer (version 1.6.20), CiteSpace (version 6.3.R1) and the R package ‘bibliometrix’ (version 4.4.1). These tools facilitated comprehensive visualization and interpretation of the data. VOSviewer was utilized to map institutional and author collaborations, co‐authorship patterns, citation networks and keyword co‐occurrence. Its ability to generate network visualizations allowed for an in‐depth exploration of relationships among key contributors and topics within the field [18]. Co‐occurrence networks of important keywords in the scientific literature were constructed and visualized using the software's embedded clustering algorithm. Co‐authorship and co‐occurrence analyses were the primary focus of this study. VOSviewer was also used to analyse the collaboration of countries, institutions and authors. The size of nodes represents the number of publications, the thickness of lines symbolizes the strength of the link and the colour of nodes stands for different clusters or times.

CiteSpace focused on identifying emerging trends and research hotspots through keyword burst detection [19]. The analysis parameters were configured as follows: the time slicing was set from 1977 to 2024, with keywords designated as the node type. Pruning was applied using the pathfinder and clip merge network methods to refine the network structure, ensuring clarity and interpretability. Bibliometrix, an R‐based package, was employed for comprehensive trend analysis, geographic distribution mapping and impact assessment. Metrics such as the h‐index, g‐index and m‐index were calculated to evaluate the academic influence of authors and journals [20, 21]. Additionally, Journal Citation Reports (JCR) quartiles and impact factors (IF) were analysed to assess journal prestige and citation influence, using the latest available data for 2023. By integrating these tools, the study systematically quantified and visualized research outputs, collaborations and thematic evolutions in the domain of CRBSIs.

## 3 Results

### 3.1 The Publication and Citation Trends

**FIGURE 1:**
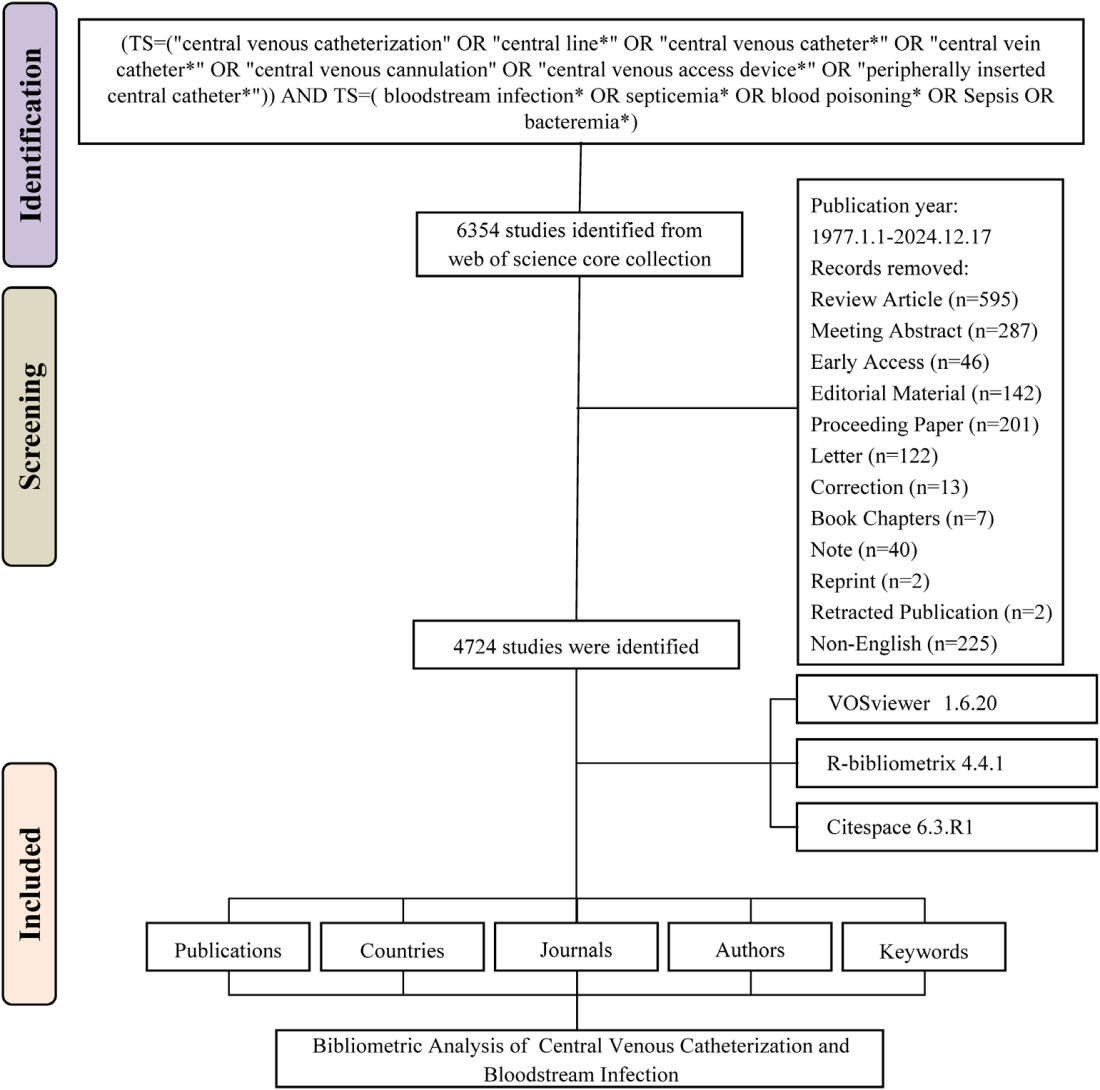
Flowchart of data screening process.

**FIGURE 2:**
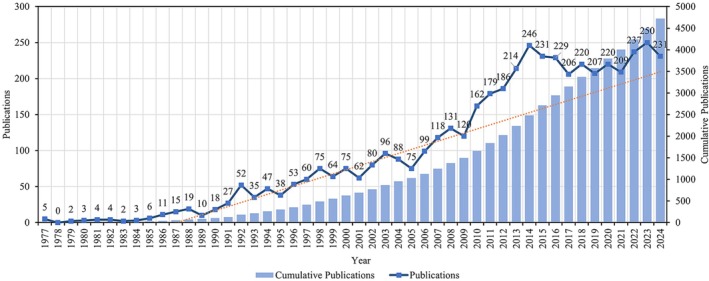
Annual number of publications from 1977 to 2024.

A detailed flowchart illustrating the literature screening process was presented in Figure 1. From 1977 to 2024, a total of 4724 documents had been published, contributed by 25 807 authors from 16 632 institutions. These works were published in 773 journals, citing 63 717 references. As shown in Figure 2, the publication trend indicated consistent growth, particularly after 2010. Before 1990, annual publications remained below 20, but significant increases were observed after 2000, reaching a peak of 250 publications in 2023. Despite slight declines in specific years, the overall trajectory remained upward.

### 3.2 Analysis of Countries

**FIGURE 3:**
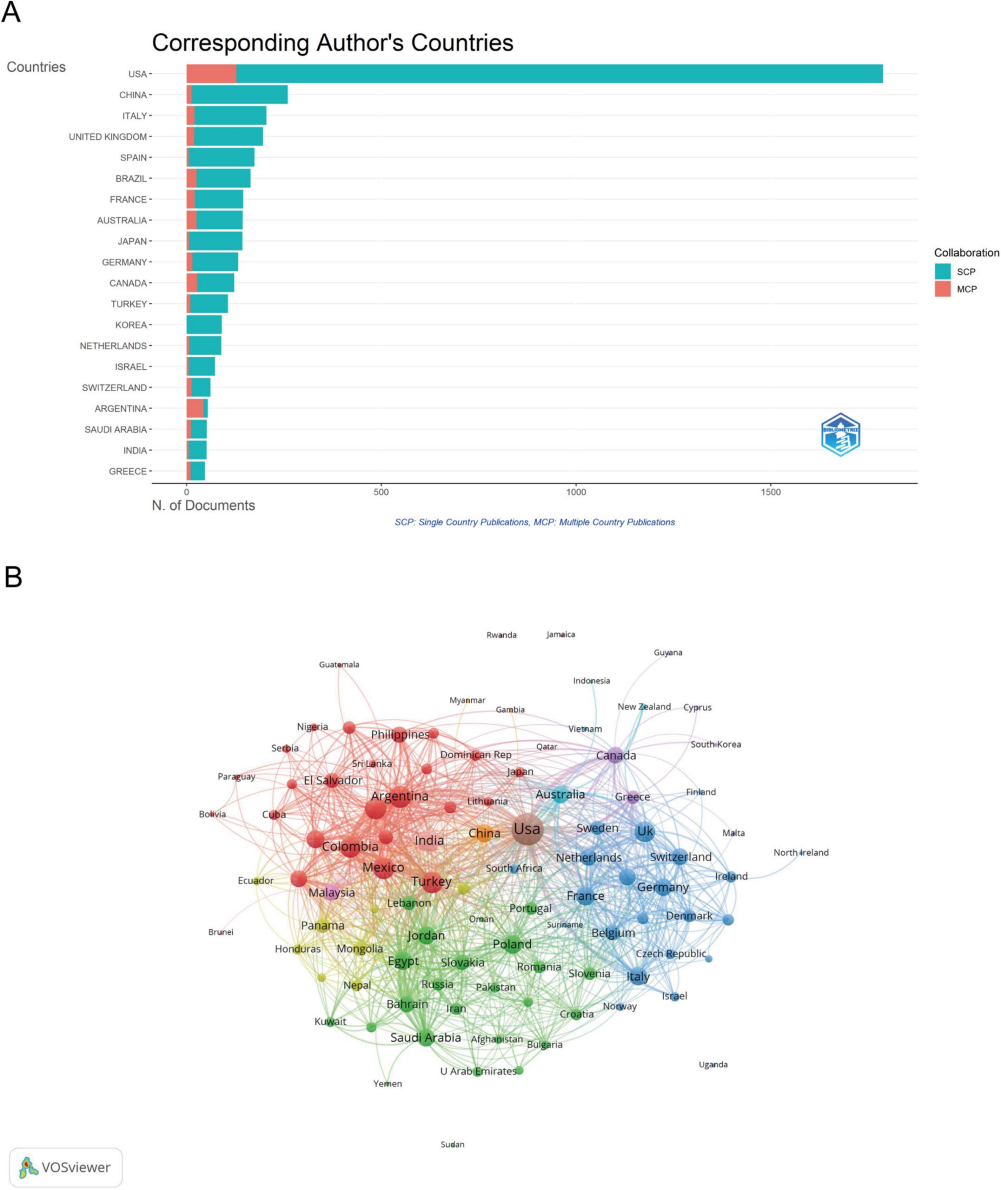
Global distribution and collaboration. (A) Distribution of corresponding authors' publications by country. (B) Visualization map depicting the collaboration among different countries.

The global distribution of publications demonstrated significant contributions from multiple countries. As shown in Table S1, the United States led with 1788 articles (37.8%), followed by China (*n* = 260, 5.5%) and Italy (*n* = 205, 4.3%). Regarding the citations, the United States occupied the leading role with 71 881 total citations (TC), followed by France (TC = 7202) and Italy (TC = 6088). Moreover, the United States contributed most multiple country publications (MCP) with 128, followed by Argentina (*n* = 43) and Canada (*n* = 27) (Figure 3A). Among the 102 countries involved in international collaborations with a minimum of 1 article, the United States (link strength = 561) had the highest number of collaborations with other countries, followed by Argentina (link strength = 220) and Mexico (link strength = 198) (Figure 3B).

### 3.3 Analysis of Institutions

**FIGURE 4:**
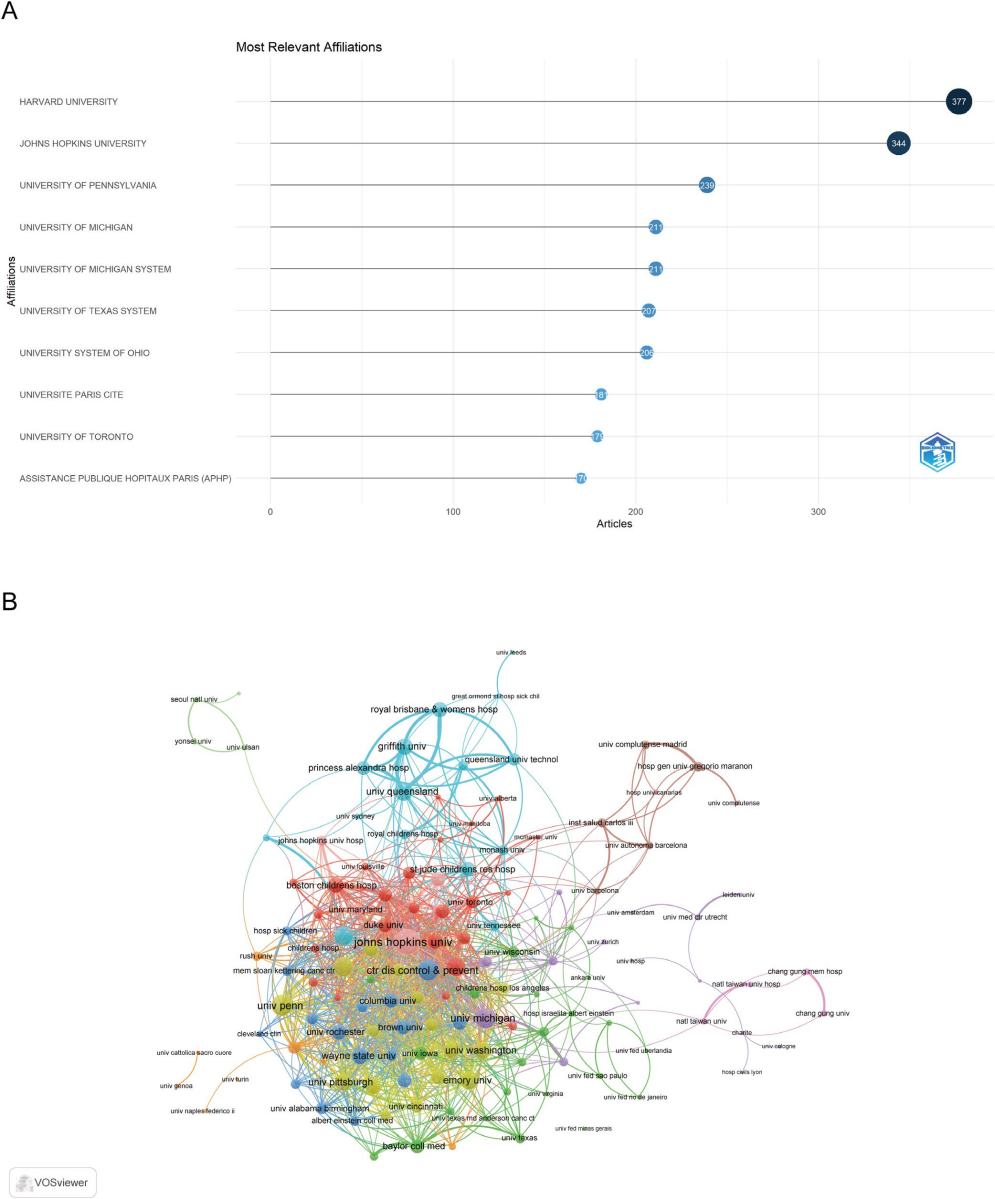
Institutional contributions and collaborations. (A) Top 10 institutions by article count and rank. (B) Visualization map depicting the collaboration among different institutions.

To explore institutions' contributions, the number of publications from various institutions was analysed. The top 10 productive institutions were displayed in Figure 4A. Harvard University ranked first with 377 publications, followed by Johns Hopkins University (*n* = 344) and the University of Pennsylvania (*n* = 239). These institutions also played pivotal roles in driving research output and collaboration within this domain. VOSviewer was used for visualization of collaboration between institutions in Figure 4B. Among the 136 institutions involved in international collaborations with a minimum of 13 articles, Johns Hopkins University had the highest number of collaborations with other institutions (link strength = 222), followed by Centers for Disease Control and Prevention (link strength = 155) and University of Pennsylvania (link strength = 149).

### 3.4 Analysis of Authors and Co‐Cited Authors

**FIGURE 5:**
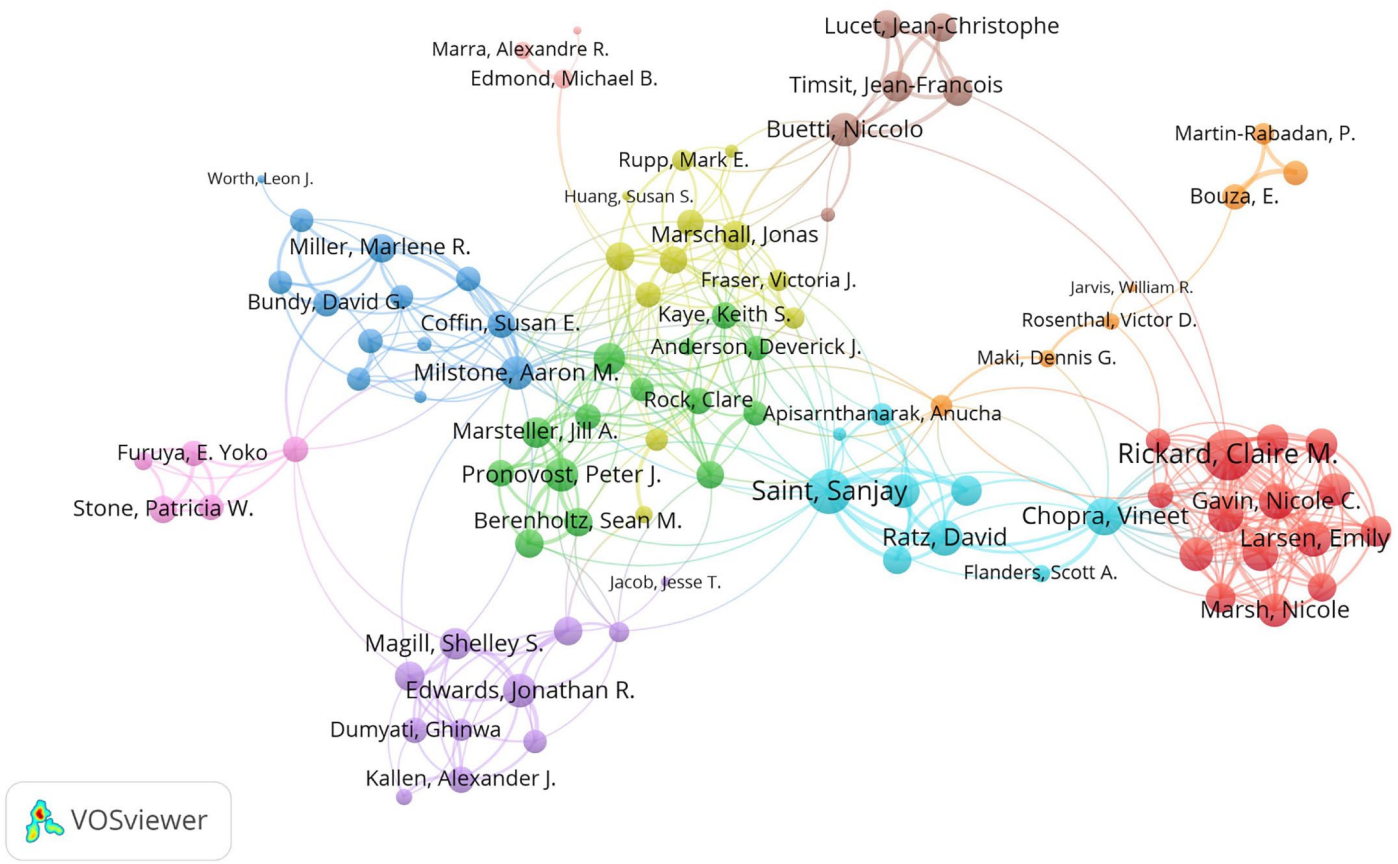
Author collaboration network.

A total of 25 807 authors had contributed to this research field, with significant variation in productivity and impact. As detailed in Table S2, Victor D. Rosenthal stood out with 61 publications and an h‐index of 34, highlighting his substantial influence. Similarly, Raad I. Issa and Claire M. Rickard also demonstrated notable contributions, with 66 and 33 publications, respectively, accompanied by h‐indexes of 31 and 14. In terms of total citations, Jonathan R. Edwards had the most citations with 7287, followed by Victor D. Rosenthal (4043) and Dennis G. Maki (3629), respectively. Among the 95 authors involved in international collaborations with a minimum of 7 articles, Claire M. Rickard had the highest number of collaborations with other authors (link strength = 90), followed by Yan Jiang (link strength = 69) and Sanjay Saint (link strength = 67), respectively (Figure 5).

### 3.5 Analysis of Journals and Co‐Cited Journals

**FIGURE 6:**
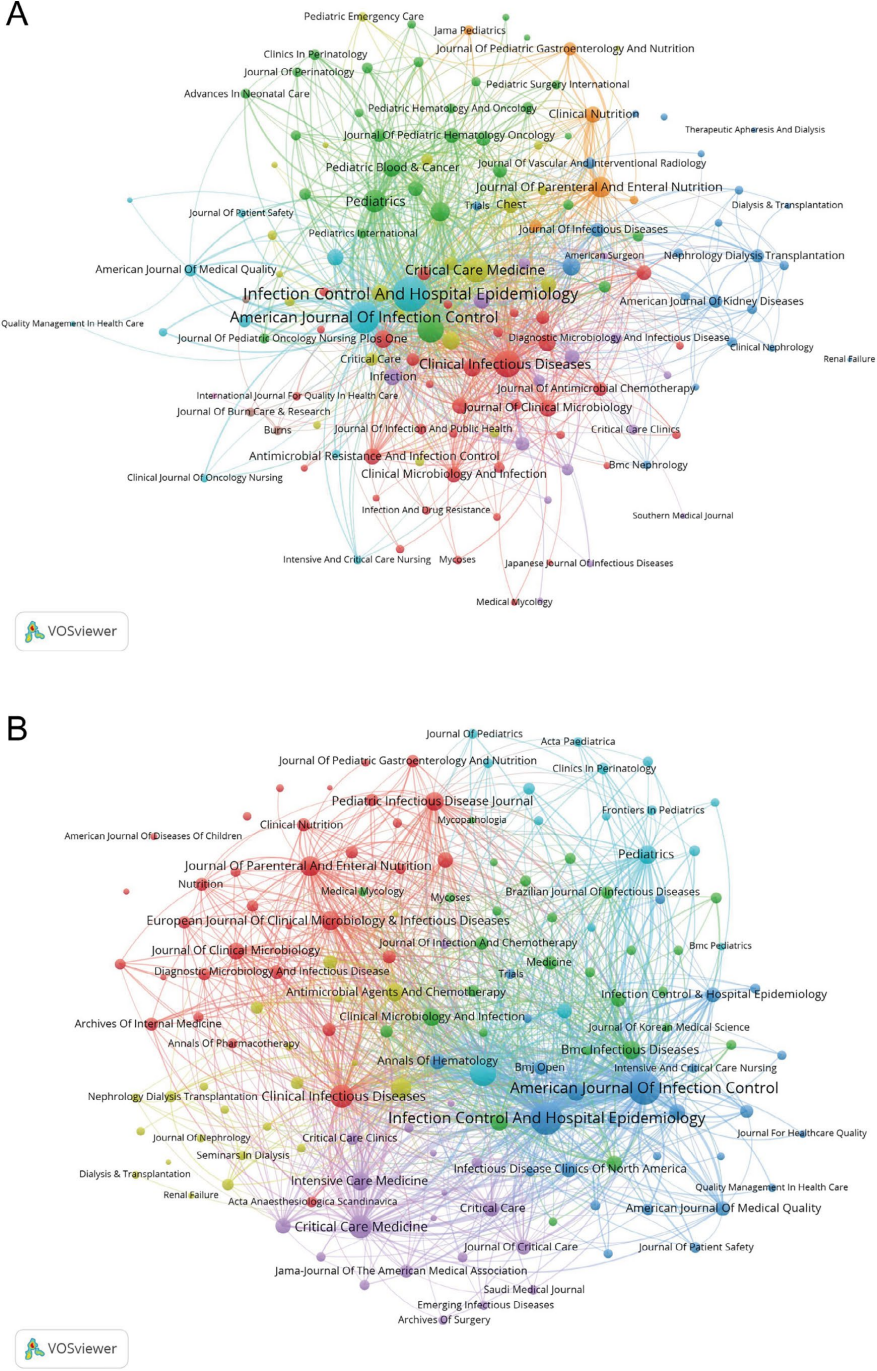
Network analyses of journals. (A) The co‐occurrence networks of journals. (B) The coupling networks of journals.

The collected papers were distributed across 773 journals, with *American Journal of Infection Control* leading in productivity with 323 publications and h‐index of 46 (Q1, IF = 3.8), followed by *Infection Control and Hospital Epidemiology* with 269 publications and h‐index of 56 (Q2, IF = 3) and the *Journal of Hospital Infection* with 148 publications and h‐index of 40 (Q1, IF = 3.9) (Table S3). In terms of total citations, *Infection Control and Hospital Epidemiology* stood out with 6875 citations, followed by *Clinical Infectious Diseases* (6679) and *American Journal of Infection Control* (5588). Co‐occurrence networks analysis, reflecting how often the publications from two different journals are associated in the bibliographies of scholarly articles [22], showed that the three key journals with the highest total link strength were *Infection Control and Hospital Epidemiology* (link strength = 3112), *American Journal of Infection Control* (link strength = 2331) and *Clinical Infectious Diseases* (link strength = 1432) (Figure 6A). Besides, coupling networks analysis was performed, capturing the degree to which the research published in two different journals relies on the same body of prior work [23], and the three key journals with the highest total link strength were *Infection Control and Hospital Epidemiology* (link strength = 148 515), *American Journal of Infection Control* (link strength = 142 617) and the *Journal of Hospital Infection* (link strength = 78 545) (Figure 6B).

### 3.6 Analysis of Co‐Occurring Keywords and Burst Terms

The analysis of keywords offered a detailed overview of research hotspots and trends in the field. The top 20 keywords with the highest occurrences were shown in Table S4.

**FIGURE 7:**
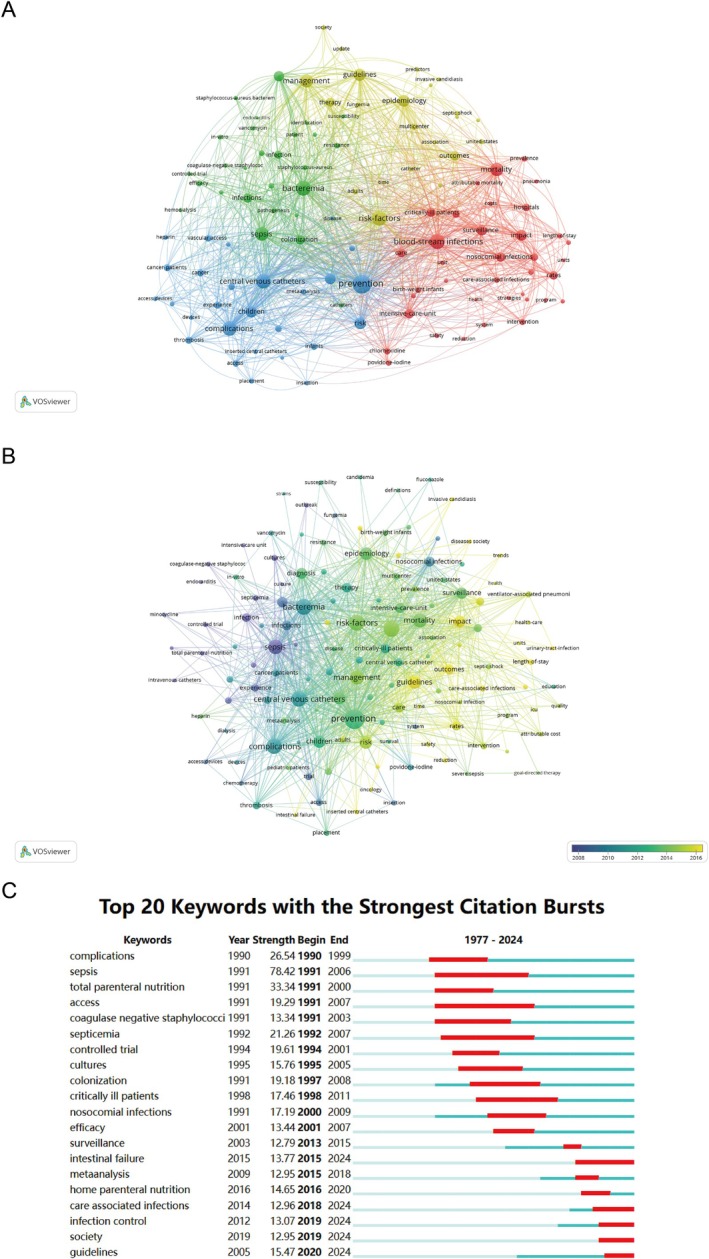
Keywords analysis. (A) Cluster analysis of keyword co‐occurrence network analysis. (B) Time‐overlapping co‐occurrence analysis network of keywords. (C) Citation burst analysis of keywords.

These keywords can be grouped into four categories (Figure 7A). The red cluster represented epidemiology and outcomes of CRBSIs, including ‘prevalence’, ‘mortality’ and ‘impact’. The green cluster centred around the treatment for CRBSIs, including ‘efficacy’, ‘controlled trial’, ‘vancomycin’ and ‘total parenteral nutrition’. The blue cluster emphasized prevention for CRBSIs, with terms like ‘prevention’ and ‘risk’. The yellow cluster highlighted the guidelines for CRBSIs, featuring keywords such as ‘guidelines’, ‘update’ and ‘multicenter’.

The time‐overlay visualization map was established in Figure 7B. Earlier studies, indicated by darker nodes, primarily explored broader themes such as ‘sepsis’, ‘complications’ and ‘outbreak’, which emphasizing the CRBSIs‐induced complications, such as sepsis. Meanwhile, recent studies had concentrated on topics such as ‘guidelines’, ‘outcomes’, ‘rates’ and ‘length‐of‐stay’, highlighting the importance in the management of CRBSIs to improve the prognosis.

In Figure 7C, the intensity of the top 20 keywords with the most notable bursts ranged from 12.79 to 78.42. Prominently, the keyword ‘access’ exhibited the most lasting burst (1991–2007) and ‘sepsis’ owned the highest burst strength with 78.42, demonstrating the severity of sepsis caused by CRBSIs. Notably, since 2019, the keywords ‘infection control’ (strength = 13.07), ‘society’ (strength = 12.95) and ‘guidelines’ (strength = 15.47) had still been prominently concentrated, indicating the critical need for standardized protocols to mitigate CRBSIs.

## 4 Discussion

### 4.1 Overview of Findings

The bibliometric assessment identifies a clear upward trend in research output related to central venous catheterization and associated bloodstream infections, with accelerated growth observed after 2010. This trend is primarily driven by the recognition of CRBSIs as a critical clinical and economic challenge. Vascular access is essential for managing critically ill, oncology and haemodialysis patients, yet it simultaneously introduces a significant risk of infection [24]. Epidemiological data from the period confirm that CRBSIs became a predominant cause of healthcare‐associated bloodstream infections, linked to considerable patient harm. For instance, U.S. studies reported more than 250 000 annual cases, with attributable mortality rates of 12%–25% in intensive care units and added costs per infection ranging from 3000 to over 56 000 [24]. The substantial burden on healthcare systems has driven heightened attention from both researchers and policymakers worldwide.

The United States has established itself as the dominant leader in this research field, evidenced not only by its exceptional volume of publications but also by its sustained academic influence, reflected in the highest total citation counts globally. This preeminence is supported by strategic research prioritization, substantial resource investment and foundational contributions to clinical guidelines. Given that CVCs are fundamental to modern oncology and critical care, the development and dissemination of evidence‐based catheter management practices are essential for improving patient outcomes. The United States has been instrumental in this regard, producing several key clinical guidelines that have helped shape international standards [25, 26, 27]. The country's scholarly impact is further reinforced by its concentration of world‐leading research institutions—such as Harvard University, Johns Hopkins University and the University of Pennsylvania—which are among the most influential globally. These institutions form extensive collaborative networks that drive innovation, consolidate expertise and accelerate the translation of research into practice, thereby maintaining the United States' central role in advancing the field. Notably, despite ranking as the second most productive country in terms of publication output, China exhibits a lower average citation count than several other leading research nations. This discrepancy may be attributed to relatively limited international collaboration, as indicated by the low proportion of internationally co‐authored papers (MCP ratio: 5%), which may reduce global visibility and citation networks. In addition, some publications may be more closely aligned with regional clinical contexts, attracting less international citation attention. The relatively recent growth of publications from China in this field may also mean that some articles have had less time to accumulate citations. At the individual level, Professor Victor D. Rosenthal of the University of Miami emerged as the most frequently cited author, a distinction attributable to his high volume of impactful publications. His work focuses on the surveillance, prevention and control of healthcare‐associated infections and multidrug‐resistant organisms. Notably, he played a leading role in a recent multinational prospective study on the incidence and risk factors for CRBSIs, the findings of which have provided critical evidence for informing both national and local health policies [28]. This aligns with the broader translational research emphasis aimed at addressing the substantial clinical burden of CRBSIs. The leading journals in the field, such as the *American Journal of Infection Control* (the official publication of the Association for Professionals in Infection Control and Epidemiology) and *Infection Control and Hospital Epidemiology* (the official publication of the Society for Healthcare Epidemiology of America), serve as vital platforms. They provide practical guidance, foster collaboration and disseminate cutting‐edge developments, thereby supporting ongoing research and clinical advancements in CRBSIs management and prevention.

### 4.2 Emerging Keywords and Research Hotspots

Based on keyword co‐occurrence analysis via VOSviewer, four interconnected research hotspots were identified, reflecting a progression from epidemiological understanding to clinical management and preventive action.

#### 4.2.1 Cluster 1 (Red): Epidemiology and Outcomes—Defining the Problem

CVCs are associated with approximately 90% of all CRBSIs, establishing them as a primary cause of this common and serious hospital‐acquired complication [29]. The incidence typically ranges from 0.5 to 5.0 cases per 1000 catheter‐days [30]. While epidemiological profiles vary globally—for instance, the average CRBSI rate in Chinese ICUs (1.5 per 1000 catheter‐days) is comparable to rates in several developed nations [31]—the aggregate patient burden remains substantial. In China, the large patient population translates into a considerable number of cases, imposing a significant financial strain on the healthcare system [4]. In the United States, hospitals contend with an estimated 80 000 CRBSIs cases annually, associated with nearly 30 000 deaths [3]. These infections consistently contribute to elevated mortality and morbidity, prolonged hospital stays and increased treatment costs [32], underscoring the urgent need for effective interventions.

#### 4.2.2 Cluster 2 (Green): Treatment—Managing Established Infection

Building on the epidemiological burden, effective treatment protocols are critical. When CRBSIs are clinically suspected, obtaining blood cultures and initiating empiric antibiotic therapy—typically covering both Gram‐positive and Gram‐negative pathogens—is essential [33]. Definitive therapy should then be tailored to culture results and patient‐specific factors [6]. Although catheter removal remains the most definitive intervention, it is not always feasible. In such cases, systemic antibiotics combined with antimicrobial lock therapy have emerged as a salvage strategy [34, 35], despite limited high‐quality evidence. Catheter removal is strongly recommended in severe or complicated infections, especially those involving high‐risk pathogens such as *S. aureus* , *Pseudomonas aeruginosa* or fungi, due to the elevated risk of treatment failure [33]. Given the complexity and suboptimal outcomes of treating established infections, the focus logically shifts toward prevention.

#### 4.2.3 Cluster 3 (Blue): Prevention—From Bundles to Adjuncts

Prevention remains paramount and is most effectively achieved through strict adherence to evidence‐based ‘bundles’. These standardized sets of interventions typically encompass hand hygiene, maximal sterile barrier precautions, chlorhexidine‐based skin antisepsis, optimal catheter site selection and the prompt removal of unnecessary catheters [33, 36, 37, 38]. While adjunctive technologies such as antibiotic or ethanol lock solutions [39, 40, 41] and antimicrobial‐impregnated catheters [42] have been investigated, their efficacy remains inconsistent and concerns regarding antimicrobial resistance limit their routine use. Notably, some randomized trials have not demonstrated significant additional benefit in reducing CRBSIs with these adjuncts [33, 43, 44]. Consequently, standardized, foundational preventive practices constitute the cornerstone of CRBSIs reduction, underscoring the need for clear, actionable clinical guidance. For bedside nurses, this translates into a non‐negotiable commitment to bundle fidelity. Each element, from hand hygiene to catheter site care, represents a nursing‐sensitive intervention whose consistent execution directly influences infection rates. Nurses' vigilance during daily line‐necessity assessments and their adherence to aseptic technique during catheter access are not merely procedural tasks but core determinants of patient safety.

#### 4.2.4 Cluster 4 (Yellow): Guidelines—Translating Evidence Into Practice

The integration of epidemiological evidence, therapeutic data and preventive approaches has led to the formulation of standardized clinical guidelines. Leading national and international organizations, including the Chinese Society of Critical Care Medicine [45], the Society for Healthcare Epidemiology of America [46] and the International Society for Infectious Diseases [47], have published updated recommendations to guide and harmonize clinical practice. These guidelines are particularly crucial in resource‐limited settings, where CRBSIs incidence rates remain substantially elevated. Their systematic implementation is indispensable for reducing the global burden of CRBSIs and improving patient outcomes, thereby completing the translational cycle from problem identification to the deployment of effective solutions. The translation of these guidelines into daily practice is fundamentally a process driven by nursing professionals. Nurses are uniquely positioned to operationalize recommendations at the point of care, audit adherence through structured monitoring and educate the broader healthcare team. A clear understanding of the evolving guideline landscape—as mapped in the present study—empowers nurses to act as both agents of change and champions for standardizing evidence‐based best practices within their clinical units.

### 4.3 Research Focus and Temporal Evolution

Early research predominantly centred on general complications associated with CRBSIs, as reflected in keywords such as ‘sepsis’, ‘complications’ and ‘outbreak’. Sepsis represents one of the most severe outcomes of CRBSIs, with CVCs posing a 64 times greater risk of sepsis compared to peripheral catheters [48]. This recognition underscored the direct link between catheter management and patient outcomes, leading to proposals to use catheter‐related sepsis incidence as a quality indicator in clinical settings. Over time, however, the research emphasis has shifted toward more structured and outcome‐oriented themes, including ‘guidelines’, ‘outcomes’, ‘rates’ and ‘length of stay’, highlighting a transition from describing the problem to systematically addressing it through evidence‐based management. Recent studies and clinical guidelines increasingly stress the importance of prevention over treatment. For instance, the European Society for Clinical Nutrition and Metabolism (ESPEN) emphasizes that preventing CRBSIs is paramount to delivering high‐quality care [49]. Similarly, guidelines from the Infectious Diseases Working Party (AGIHO) of the German Society of Hematology and Medical Oncology (DGHO) provide structured recommendations on catheter removal, antimicrobial lock therapy (ALT) and systemic antibiotic treatment, reinforcing the need for protocol‐driven interventions [50, 51]. This evolution reflects a broader trend toward standardization and guideline implementation in clinical practice.

### 4.4 Emerging Directions and Future Perspectives

In the past 5 years (2019–2024), several high‐impact keywords have emerged, signalling new research priorities and methodological advances. The term ‘infection control’ has gained prominence, reflecting intensified efforts to implement standardized, evidence‐based interventions to reduce healthcare‐associated infections. A landmark study in this area is the LOCK IT‐100 trial, a phase 3 randomized controlled trial that demonstrated a 71% reduction in CRBSIs risk with taurolidine/heparin lock solution compared to heparin alone in haemodialysis patients with CVCs [52]. This trial not only validates the efficacy of advanced lock solutions but also highlights the growing role of rigorous, multicentre studies in shaping preventive strategies. Concurrently, the development and updating of clinical ‘guidelines’ by professional societies—such as the Spanish Society of Infectious Diseases and Clinical Microbiology (SEIMC) and the Spanish Society of Intensive and Critical Care Medicine and Coronary Units (SEMICYUC) [53]—illustrate an ongoing commitment to harmonizing global practices. As medical technology advances, the need for continuously updated, context‐specific guidelines will persist, guiding healthcare workers worldwide in the standardized management of CRBSIs. Looking forward, nursing research and quality improvement projects should focus on: (1) implementing and auditing preventive bundles in diverse ICU settings, (2) evaluating nurse‐led interventions such as structured patient education programmes or novel dressing protocols, and (3) integrating nurse‐driven surveillance data into digital health tools. This positions nursing at the forefront of the proactive, precision‐based management of CRBSIs. These directions collectively point toward a more proactive, precision‐based and systematically managed approach to reducing the burden of CRBSIs globally.

### 4.5 Strengths and Limitations

This bibliometric study provides valuable insights into the evolving research landscape of CRBSIs. By mapping influential contributors and emerging thematic trends, it offers a strategic roadmap for researchers to identify priority areas and foster collaborative partnerships. The analysis of recent burst keywords further helps delineate cutting‐edge topics, such as infection control and clinical guidelines, which are likely to direct future scientific inquiry. Nevertheless, this study has certain limitations. First, while the WoSCC provides a well‐established foundation for citation analysis, its exclusive use in this study may have led to the omission of nursing‐specific literature and international clinical trials that are indexed in other databases such as Scopus or PubMed/MEDLINE. This could introduce a selection bias, particularly in underrepresenting regionally important studies or studies published in specialized nursing journals, and may limit the generalizability of the findings to the broader global evidence base. Second, the restriction to English‐language publications may introduce linguistic and geographical bias. The screening process (Figure 1) excluded 225 non‐English records, primarily in Chinese, Spanish or Portuguese, languages predominant in high‐burden regions such as Asia and Latin America. Consequently, the ‘global’ research map presented here may not fully capture the diversity of evidence and perspectives from these regions, and this limitation should be considered when interpreting the findings. Finally, the exclusion of non‐original publications, including review articles, books, editorials and conference proceedings, may have narrowed the scope of the analysis and led to the omission of influential synthesizing works. Future studies would benefit from incorporating multiple databases and a wider variety of document types to enable a more holistic understanding of research developments in this field.

## 5 Conclusions

This study employed bibliometric methods to evaluate research trends in CRBSIs, analysing publication outputs, collaboration networks and thematic keywords. An upward trajectory in publication output, particularly after 2010, may be due to the popularization of vascular catheters in critically ill and haemodialysis patients. The research hotspots focus on epidemiology and outcomes, treatment, prevention and guidelines for CRBSIs. Clinically, future research should focus on the establishment of advanced guidelines by professional societies based on high‐quality trials.

## Author Contributions

Conception and design: Jianrong Li and Zhonghua Li. Administrative support: Junyu Zhu. Data analysis and interpretation: Leping Wang and Xuefang Liu. Manuscript writing: Jianrong Li, Zhonghua Li, Junyu Zhu, Leping Wang, and Xuefang Liu. Final approval of manuscript: Jianrong Li, Zhonghua Li, Junyu Zhu, Leping Wang, and Xuefang Liu.

## Funding

This work was supported by the Medical Science Research Project of Hebei (20242308).

## Ethics Statement

The authors have nothing to report.

## Consent

The authors have nothing to report.

## Conflicts of Interest

The authors declare no conflicts of interest.

## Supporting information


**Table S1:** Publication and citation profiles of leading countries.
**Table S2:** Publication and citation profiles of high‐impact authors.
**Table S3:** Bibliometric indicators of high‐impact journals.
**Table S4:** Top 20 keyword co‐occurrence network analysis.

## Data Availability

All data generated or analysed during this study are included in this published article.
