# Supplementary material for: The Hotspots and Publication Trends in Central Venous Catheter‐Related Bloodstream Infection: A Bibliometric Analysis
Source: Nurs Crit Care. 2026 Aug 6;31(5):e70596. doi: 10.1111/nicc.70596 (PMC13444666; doi:10.1111/nicc.70596)
Supplement: Supplementary file 1 [file NICC-31-0-s001.docx]

**Table S1 Publication and citation profiles of leading countries**

| **Country** | **Articles** | **Freq** | **SCP** | **MCP** | **MCP_Ratio** | **TP** | **TP_rank** | **TC** | **TC_rank** | **Average Citations** |
| --- | --- | --- | --- | --- | --- | --- | --- | --- | --- | --- |
| United States | 1788 | 37.8 | 1660 | 128 | 7.2 | 6754 | 1 | 71881 | 1 | 40.2 |
| China | 260 | 5.5 | 247 | 13 | 5 | 944 | 2 | 4112 | 7 | 15.8 |
| Italy | 205 | 4.3 | 185 | 20 | 9.8 | 811 | 5 | 6088 | 3 | 29.7 |
| Uk | 196 | 4.1 | 177 | 19 | 9.7 | 691 | 7 | 5927 | 4 | 30.2 |
| Spain | 174 | 3.7 | 169 | 5 | 2.9 | 842 | 3 | 5788 | 5 | 33.3 |
| Brazil | 164 | 3.5 | 139 | 25 | 15.2 | 668 | 8 | 3823 | 9 | 23.3 |
| France | 145 | 3.1 | 124 | 21 | 14.5 | 767 | 6 | 7202 | 2 | 49.7 |
| Australia | 144 | 3 | 119 | 25 | 17.4 | 823 | 4 | 3529 | 10 | 24.5 |
| Japan | 143 | 3 | 137 | 6 | 4.2 | 448 | 11 | 1535 | 16 | 10.7 |
| Germany | 132 | 2.8 | 117 | 15 | 11.4 | 519 | 10 | 4264 | 6 | 32.3 |
| Canada | 122 | 2.6 | 95 | 27 | 22.1 | 587 | 9 | 3351 | 11 | 27.5 |
| Turkey | 106 | 2.2 | 97 | 9 | 8.5 | 440 | 12 | 1486 | 17 | 14 |
| Korea | 90 | 1.9 | 90 | 0 | 0 | 427 | 13 | 1537 | 15 | 17.1 |
| Netherlands | 89 | 1.9 | 82 | 7 | 7.9 | 372 | 15 | 2073 | 13 | 23.3 |
| Israel | 73 | 1.5 | 68 | 5 | 6.8 | 354 | 16 | 1834 | 14 | 25.1 |
| Switzerland | 61 | 1.3 | 49 | 12 | 19.7 | 255 | 17 | 2124 | 12 | 34.8 |
| Argentina | 54 | 1.1 | 11 | 43 | 79.6 | 139 | 20 | 3890 | 8 | 72 |
| Saudi Arabia | 52 | 1.1 | 41 | 11 | 21.2 | 201 | 18 | 917 | 21 | 17.6 |
| India | 51 | 1.1 | 46 | 5 | 9.8 | 377 | 14 | 609 | 22 | 11.9 |
| Greece | 47 | 1 | 37 | 10 | 21.3 | 182 | 19 | 979 | 20 | 20.8 |

Note(s): Articles: Publications of Corresponding Authors only. Freq: Frequence of Total Publications. SCP: Single Country Publications. MCP: Multiple Country Publications. MCP_Ratio: Proportion of Multiple Country Publications. TP: Total Publications. TP_rank: Rank of Total Publications. TC: Total Citations. TC_rank: Rank of Total Citations. Average Citations: The average number of citations per publication.

**Table S2 Publication and citation profiles of high-impact authors**

| **Author** | **h_index** | **g-index** | **m-index** | **PY_start** | **TP** | **TP_Frac** | **TP_rank** | **TC** | **TC_rank** |
| --- | --- | --- | --- | --- | --- | --- | --- | --- | --- |
| Victor D. Rosenthal | 34 | 61 | 1.417 | 2003 | 61 | 7.65 | 2 | 1060 | 1 |
| Raad I. Issa | 31 | 66 | 0.886 | 1992 | 66 | 13.14 | 1 | 1007 | 2 |
| Jonathan R. Edwards | 22 | 29 | 0.759 | 1998 | 29 | 4.3 | 7 | 450 | 7 |
| Dennis G. Maki | 21 | 21 | 0.525 | 1987 | 21 | 4.26 | 18 | 785 | 4 |
| Sanjay Saint | 20 | 32 | 0.714 | 1999 | 32 | 5.2 | 6 | 420 | 8 |
| Ray Hachem | 18 | 32 | 0.581 | 1996 | 32 | 4.72 | 5 | 354 | 12 |
| William R. Jarvis | 18 | 21 | 0.529 | 1993 | 21 | 3.26 | 17 | 333 | 14 |
| Victoria J. Fraser | 17 | 17 | 0.654 | 2001 | 17 | 3.07 | 25 | 527 | 5 |
| Yan Jiang | 17 | 29 | 0.85 | 2007 | 29 | 3.8 | 8 | 186 | 56 |
| Peter J. Pronovost | 17 | 24 | 0.739 | 2004 | 24 | 4.49 | 13 | 331 | 15 |
| Scott K. Fridkin | 16 | 21 | 0.516 | 1996 | 21 | 2.96 | 15 | 253 | 26 |
| Petra Gastmeier | 16 | 25 | 0.552 | 1998 | 25 | 4.67 | 12 | 167 | 78 |
| Anne-Marie Chaftari | 15 | 24 | 0.833 | 2009 | 28 | 3.88 | 9 | 167 | 77 |
| Thomas S.J. Elliott | 15 | 21 | 0.441 | 1993 | 21 | 5.83 | 14 | 83 | 519 |
| Leonard A. Mermel | 15 | 21 | 0.455 | 1994 | 21 | 3.5 | 19 | 792 | 3 |
| Aaron M. Milstone | 15 | 20 | 0.882 | 2010 | 20 | 2.8 | 21 | 261 | 25 |
| Vineet Chopra | 14 | 25 | 1 | 2013 | 25 | 3.83 | 11 | 215 | 41 |
| Claire M. Rickard | 14 | 23 | 0.609 | 2004 | 33 | 4.48 | 3 | 104 | 321 |
| Christine Geffers | 13 | 21 | 0.481 | 2000 | 21 | 4 | 16 | 156 | 101 |
| Peter Kite | 13 | 13 | 0.419 | 1996 | 13 | 2.53 | 51 | 162 | 90 |

Note(s): h-index indicates the author’s productivity and citation impact. g-index gives greater weight to highly cited publications by the author. m-index is the h-index divided by the number of years since the author’s first publication in this field. PY_start indicates the first publication year of the author in this field. TP = total publications. TP_Frac = fractionalized total publications. TP_rank = rank of total publications. TC = total citations. TC_rank = rank of total citations.

**Table S3 Bibliometric indicators of high-impact journals**

| **Journal** | **H_index** | **G_index** | **M_index** | **IF 2023** | **JCR 2023** | **TP** | **TP_rank** | **TC** | **TC_rank** | **PY_start** |
| --- | --- | --- | --- | --- | --- | --- | --- | --- | --- | --- |
| Infection Control and Hospital Epidemiology | 56 | 116 | 1.647 | 3 | 2 | 269 | 2 | 6875 | 1 | 1991 |
| Clinical Infectious Diseases | 51 | 98 | 1.545 | 8.2 | 1 | 102 | 4 | 6679 | 2 | 1992 |
| Critical Care Medicine | 47 | 75 | 1.424 | 7.7 | 1 | 75 | 8 | 3946 | 4 | 1992 |
| American Journal of Infection Control | 46 | 74 | 1.07 | 3.8 | 1 | 323 | 1 | 5588 | 3 | 1982 |
| Journal of Hospital Infection | 40 | 61 | 1.081 | 3.9 | 1 | 148 | 3 | 3027 | 6 | 1988 |
| Pediatrics | 40 | 60 | 1.026 | 6.2 | 1 | 60 | 9 | 2728 | 7 | 1986 |
| Pediatric Infectious Disease Journal | 33 | 50 | 0.868 | 2.9 | 1 | 87 | 6 | 1599 | 14 | 1987 |
| Journal of Clinical Microbiology | 32 | 51 | 0.821 | 6.1 | 1 | 51 | 13 | 2725 | 8 | 1986 |
| Journal of Parenteral and Enteral Nutrition | 29 | 44 | 0.707 | 3.2 | 2 | 97 | 5 | 1600 | 13 | 1984 |
| PLOS ONE | 24 | 40 | 1.6 | 2.9 | 1 | 52 | 12 | 828 | 27 | 2010 |
| Intensive Care Medicine | 23 | 42 | 0.622 | 27.1 | 1 | 42 | 14 | 1624 | 12 | 1988 |
| BMC Infectious Diseases | 22 | 34 | 1.158 | 3.4 | 2 | 57 | 10 | 515 | 38 | 2006 |
| Chest | 22 | 31 | 0.647 | 9.5 | 1 | 31 | 24 | 1243 | 20 | 1991 |
| Clinical Microbiology and Infection | 22 | 38 | 0.917 | 10.9 | 1 | 38 | 20 | 1306 | 18 | 2001 |
| European Journal of Clinical Microbiology & Infectious Diseases | 22 | 35 | 0.55 | 3.7 | 2 | 57 | 11 | 1126 | 22 | 1985 |
| Infection | 21 | 34 | 0.512 | 5.4 | 1 | 41 | 15 | 625 | 32 | 1984 |
| Clinical Nutrition | 20 | 39 | 0.513 | 6.6 | 1 | 39 | 19 | 682 | 30 | 1986 |
| Antimicrobial Agents and Chemotherapy | 19 | 40 | 0.731 | 4.1 | 1 | 40 | 17 | 1691 | 11 | 1999 |
| Journal of Antimicrobial Chemotherapy | 19 | 25 | 0.594 | 3.9 | 1 | 25 | 31 | 1328 | 17 | 1993 |
| Pediatric Critical Care Medicine | 19 | 28 | 1.056 | 4 | 1 | 32 | 23 | 398 | 51 | 2007 |

Note(s): h_index: The h-index of the journal, which measures both the productivity and citation impact of the publications. g_index: The g-index of the journal, which gives more weight to highly-cited articles. m_index: The m-index of the journal, which is the h-index divided by the number of years since the first published paper. IF_2023: Impact Factor in 2023, indicating the average number of citations to recent articles published in the journal. JCR_2023: The quartile ranking of the journal in the Journal Citation Reports in 2023, indicating the journal's ranking relative to others in the same field (Q1: top 25%, Q2: 25%-50%, Q3: 50%-75%, Q4: bottom 25%). TP: Total Publications. TP_rank: Rank of Total Publications. TC: Total Citations. TC_rank: Rank of Total Citations. PY_start: Publication Year Start, indicating the year the journal started publication.

**Table S4** **Top 20 keyword co-occurrence network analysis**

| id | keyword | occurrences | total link strength |
| --- | --- | --- | --- |
| 3106 | prevention | 852 | 3666 |
| 375 | bacteremia | 630 | 2417 |
| 3416 | risk-factors | 558 | 2411 |
| 464 | blood-stream infections | 572 | 2349 |
| 894 | complications | 532 | 2272 |
| 686 | central venous catheters | 526 | 2223 |
| 3510 | sepsis | 500 | 1951 |
| 2342 | management | 436 | 1807 |
| 2485 | mortality | 422 | 1797 |
| 1648 | guidelines | 361 | 1609 |
| 3410 | risk | 377 | 1489 |
| 1322 | epidemiology | 332 | 1462 |
| 726 | children | 348 | 1380 |
| 1890 | impact | 325 | 1281 |
| 463 | blood-stream infection | 310 | 1272 |
| 3800 | surveillance | 271 | 1180 |
| 1113 | diagnosis | 258 | 1119 |
| 862 | colonization | 211 | 980 |
| 2787 | outcomes | 227 | 934 |
| 2032 | intensive-care-unit | 225 | 932 |
